# Preparation of *Teucrium polium* extract-loaded chitosan-sodium lauryl sulfate beads and chitosan-alginate films for wound dressing application

**DOI:** 10.3934/publichealth.2021059

**Published:** 2021-10-29

**Authors:** Mariem Kharroubi, Fatima Bellali, Abdelhafid Karrat, Mohamed Bouchdoug, Abderrahim Jaouad

**Affiliations:** 1 Laboratory of Biotechnologies, Specialized Center of Valorization and Technology of Sea Products, National Institute of Fisheries Research (INRH), Agadir, Morocco; 2 Laboratory of Biological Engineering, Faculty of Science and Technology, Beni Mellal University Sultan Moulay Slimane, Morocco; 3 Research Team of Innovation and Sustainable Development & Expertise in Green Chemistry, “ERIDDECV”, Department of Chemistry, Cadi Ayyad University, Marrakesh, Morocco

**Keywords:** chitosan-sodium lauryl sulfate beads, chitosan-alginate films, characterization, *Teucrium polium*, wound dressing, wound healing

## Abstract

This study aimed to formulate sodium lauryl sulfate cross-linked chitosan beads and sodium alginate-chitosan films for designing a dressing that would shorten the healing time of skin wounds. *Teucrium polium* extract-loaded chitosan-sodium lauryl sulfate beads (CH-SLS) and chitosan-alginate (CH-ALG) films were prepared and characterized by using Fourier transform infrared spectroscopy (FT-IR), X-ray diffraction (XRD) analysis, and scanning electron microscopy (SEM). The swelling properties of the CH-SLS beads were also analyzed in a water solution. The obtained *Teucrium polium* extract-loaded CH-SLS beads and CH-ALG films (TBF) were further incorporated into the commercial adhesive dressing. This TBF wound dressing was then investigated for evaluation of its wound healing potential in the mice using the excision wound model. Healing was assessed by the macroscopic appearance and the rate of wound contraction during 8 days. On day 4, the TBF-treated wounds exhibited 98% reduction in the wound area when they were compared with healing ointment, elastic adhesive dressing, and untreated wounds which were exhibited 63%, 43%, and 32%, respectively. Furthermore, the application of TBF dressing reduced skin wound rank scores and increased the percentage of wounds contraction. These results demonstrate that TBF dressing improves considerably the healing rate and the macroscopic wound appearance at a short delay and this application may have therapeutic benefits in wound healing.

## 1. Introduction

According to the Wound Healing Society, woundheal.org, a wound is defined as an abnormal interruption of the anatomical structure and physiological structure of a tissue. It represents a danger for natural defense barriers, making tissues more accessible to microorganisms' invasion. Wound healing is defined by the Wound healing Society as a complex and dynamic process that restores functions and anatomical continuity tissues. Non-healing wounds are strongly correlated with high morbidity and might considerably affect a patient's well-being and economic status [1].

Recently, advanced dressings of bioactive agents are used to shorten wound healing time [2]. Chitosan (CH) is a healing agent and a material of choice for tissue engineering [3],[4]. CH hydrogels have been widely studied and their healing capacity has been demonstrated *in vivo* [5],[6] and *in vitro* studies [7],[8]. CH has the ability to stimulate healing wound by activating inflammatory cells such as macrophages [9], polymorphonuclear, and also fibroblasts, and endothelial cells [10]. In addition, one of the many advantages of CH in medical devices is its capacity to be antibacterial on (G+) bacteria (Ex Staphylococcus aureus) and (G−) (eg *Pseudomonas aeruginosa, Escherichia coli*) [11],[12]. Extensive literature revealed the alginate (ALG) is a wound healing agent [13]. They stimulate active molecules from key cells involved in the healing process [14]–[18]. ALG has also a strong absorption capacity for water. They are able to absorb water up to 300 times their own weight [19]. This is why ALG compresses are used for exuding wounds.

In addition, numerous plants and plant-derived products have been confirmed to enhance wound healing in different pharmacological models and patients. Many *in vivo* and *in vitro* investigations have demonstrated different biological activities of *Teucrium polium* such as anti-inflammatory [20] and antimicrobial [21],[22] properties. However, a few studies have shown that *Teucrium polium* is effective at wound healing [23]–[25].

In this regard, the present study was undertaken in order to evaluate the suitability of using sodium lauryl sulfate cross-linked chitosan beads with encapsulated *Teucrium polium* extract and chitosan-alginate films for designing a porous dressing that would improve wound healing. First, the chitosan-sodium lauryl sulfate beads (CH-SLS) and chitosan-alginate films (CH-ALG) were prepared and characterized using Fourier transform infrared spectroscopy (FT-IR), X-ray diffraction (XRD) analysis, and scanning electron microscopy (SEM). Secondly, the *Teucrium polium* extract-loaded CH-SLS beads and CH-ALG films (TBF) were investigated for their healing efficacy using a mice model. To our knowledge, this is the first report on the use of *Teucrium polium* extract-loaded chitosan-sodium lauryl sulfate beads (CH-SLS) and chitosan-alginate (CH-ALG) films as a wound dressing in an excision model in mice.

## 2. Materials and methods

### 2.1. Materials

Chitosan chips derived from shrimp shell was purchased from Sigma Aldrich, average molecular weight, deacetylation degree, DD = 75–85%, and dynamic viscosity 200–800 cPoise (1% in aqueous solution 1% acetic acid, V/V) was used without further purification. Medium viscosity (viscosity of 2% solution 3500 cps at 25 1C) sodium alginate from the brown algae was obtained from Sigma Chemical. Medical grade ethanol was locally obtained and was distilled before it was used. The other reagents used were analytical grade namely, acetic acid, sodium lauryl sulfate, phenolphthalein, gallic acid hydrate, CaCO_3_, and Folin-Ciocalteu reagent. Elastic adhesive bandage was obtained from Johnson & Johnson Ltd. Healing Ointment for lesion, acute and chronic wounds 30g, Cicasol ointment was bought from the pharmacy in Morocco.

### 2.2. Plant material and preparation of phenolic extract

The aerial parts of *Teucrium polium* were collected from Meknès-Tafilalet, Morocco. It was shade-dried and powdered. The dried plant material was subjected of phenolic compounds extraction by maceration according to the method of Hammoudi et al. [26]. The extract was filtered by vacuum filtration and was stored for further use.

### 2.3. Solubility test of chitosan

The solubility of CH was evaluated in acetic acid at different concentrations (0.01, 0.02, 0.05, and 0.1 mol/L). The results are summarized in Table 1. It was found that the CH was dissolved in the acetic acid at concentrations higher than 0.02 M. For this study, we chose a concentration of 0.02 M to remain within the framework of a rational optimization of the reagents for future industrial use.

**Table 1.** Observation of the different concentration acetic acid solubility of chitosan.

Concentration of acetic acid (mol/L)	Solubility
0.01	Poorly soluble
0.02	Soluble
0.05	Very soluble
0.5	Very soluble

### 2.4. Preparation of CH-SLS beads

**Figure 1.**
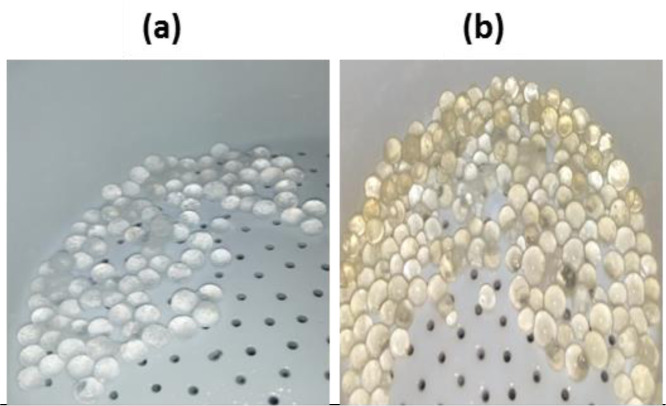
Photograph of sodium lauryl sulfate cross-linked chitosan hydrogels at filtration step before (a) and after (b) encapsulation of plant extract from *Teucrium polium*.

**Figure 2.**
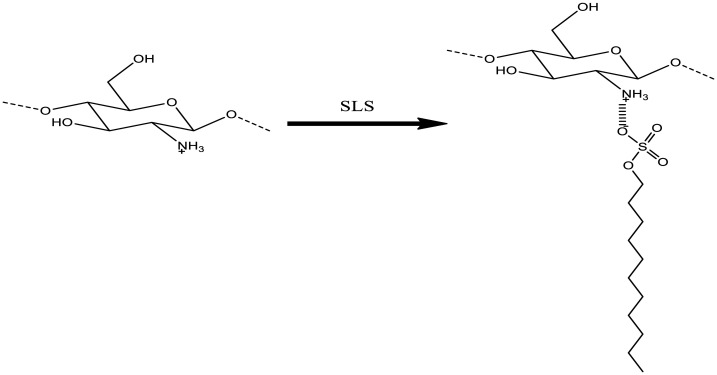
Reaction mechanism of chitosan with SLS.

The crosslinking formation was prepared by the anion interactions between the positively charged functional groups of chitosan and the sulfate anions of sodium lauryl sulfate (Figure 2). A clear solution of CH was prepared by dissolving the proper amount of CH in aqueous solutions of acetic acid (0.25% w/v in 0.02 M acetic acid). The viscous CH solution obtained was added drop by drop using a 25 mL syringe in 300 mL of 0.05 M sodium lauryl sulfate (SLS). After 30 minutes, the hydrogel is obtained. The physically crosslinked network has junctions resulting from ionic interaction between the ammonium group of chitosan and the lauryl sulfate anion. The physical hydrogel was filtered by vacuum aspiration. The obtained spherical beads were washed with distilled water until they reached neutrality. The filtrate neutrality was checked by phenolphthalein. The resultant hydrogels were freeze-dried at −45°C to generate a porous structure. 5 mg of the developed CH-SLS beads was introduced in 5 mL of the *Tecruim poluim* extract to adsorb (total phenolic content in beads 61.25 ± 0.78 mg GAE/g) the polyphenol active principles by forming hydrogen bonds with the beads based on chitosan (Figure 1).

### 2.5. Beads average diameter, water content and total phenolic content (TPC)

Particle diameters of the chitosan beads were tested (n = 20) with a digital vernier caliper (minimal distance = 0.01 mm). Water contents of the beads were measured using an infrared moisture analyzer (AD-4714A) at 105°C. For total phenolic content 20 chitosan beads were placed in test tubes with 5 mL of distilled water under continuous agitation during 15 min. After centrifugation at 5000, during 15 min, The TPC was determined in supernatant using Folin–Ciocalteu method as described in Zannou and Koca [27]. The absorbance was read at 760 nm using a UV-spectrometer (Jenway). TPC was estimated by using a gallic acid calibration curve (0–120 mg/mL). The results were expressed as gallic acid equivalents (mg GAE/g of beads).

### 2.6. Swelling test

The swelling studies of chitosan beads were carried out in distilled water at room temperature. The percentage of swelling were calculated by using the following Eq 1, where WS is the weight of swollen beads (g) and W is the weight of dry beads (g).

$$\begin{array}{c} Percentage\;of\;swelling\;\left(\%\right)=\frac{WS-W}{W}\times 100 \end{array}$$ (1)

### 2.7. TPC release studies

The total phenolic content release experiments were carried out in an alkaline medium (distilled water adjusted with 0.1 M NaOH to obtain a pH of 7.4 at 37°C according to Belscak-Cvitanovic et al. [28] with some modifications. Known amount of beads containing the *Teucrium polium* extract were placed in 30 mL of the release medium. Once every 30 min, a portion of 2 mL of solution supernatant was sampled out from the mixture individually for analysis with replacing the same portion of fresh media. The amount of total phenolic content in the samples was assessed, using a UV-Visible Spectrophotometer (JENWAY, Genova plus). The releases of beads were calculated as a percentage value respectively [29]. The percentage of released polyphenols was calculated as follows Eq 2, where (M_t_/M_∞_) represents the fraction of mass released at time t (M_t_) with respect to the maximum mass of polyphenols that would be released at time t = ∞. The experiments were performed in triplicate.

$$\begin{array}{c} Released\;polyphenols\;\left(\text{\%}\right)=\frac{\text{Mt}}{\text{M}\infty }*100 \end{array}$$ (2)

### 2.8. Preparation of CH-ALG films

**Figure 3.**
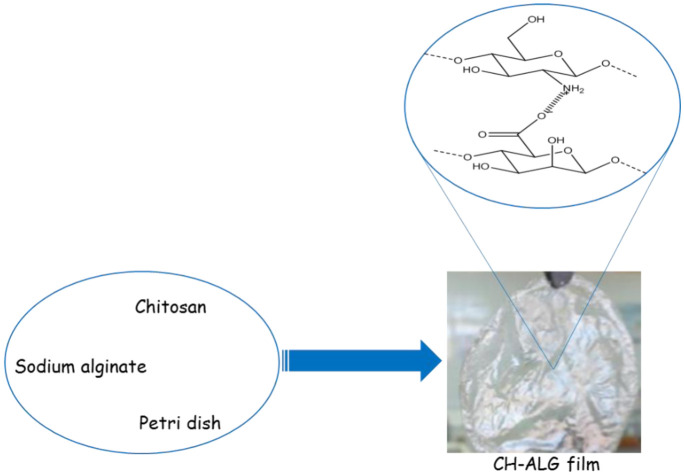
A schematic representation for the preparation of CH-ALG film.

The CH solution was prepared by dispersing 0.25 g of CH in 100 mL of acetic acid (0.02 M). The viscous CH solution was added drop by drop using a 25 mL syringe in 25 mL of sodium alginate (ALG). Then, the reaction mixture was left for 20 min with vigorous stirring. The obtained solution was poured into a polyethylene petri dish and left to dry overnight at 25°C. The obtained films were immersed in 50 mL of distilled water for 1 h to remove the unreacted components, washed three times with distilled water, and dried at 30°C under vacuum. Then, the films were packed separately and sealed in plastic bags using aseptic techniques. The packed sterilized films were then kept at room temperature in a dry and a clean place until use. A schematic representation of the CH-ALG film preparation is shown in Figure 3.

### 2.9. Film thickness, mechanical properties of CH-ALG films, and biodegradability

Film thickness was conducted with an electronic digital micrometer (Nobel) and replicated five times. The measurements were taken at five different locations of the film sample and the mean values were calculated. Regarding mechanical testing of films, the tensile strength (TS), and elongation at break (E) were carried out at room temperature and were studied with an IMADA type HV-1000N tensile machine. Five replications were conducted. Regarding biodegradation testing, films were submitted to degradation under indoor soil burial conditions for 14 days according to Martucci and Ruseckaite [30]. Biodegradation of film was studied by monitoring weight loss on an analytical balance. The average weight loss (%WL) was quantified by the following Eq 3, where M_0_ and M_t_ are the initial and the residual mass at time = t, respectively. The experiments were performed in triplicate.

$$\begin{array}{c} \%WL=\frac{\left(\text{M}0-\text{Mt}\right)}{\text{M}0}*100 \end{array}$$ (3)

### 2.10. Fourier transform infrared spectroscopy (FT-IR)

FT-IR was used to characterize the presence of specific chemical groups in the CH-SLS beads and CH-ALG films and analyzed by FT-IR using VERTEX 70 model. FT-IR spectra were obtained in the wavenumber range from 400 to 4000 cm^−1^ during 32 scans, with 4 cm^−1^ resolution.

### 2.11. X-ray diffraction (XRD)

XRD patterns of CH-SLS beads and CH-ALG films were analyzed using an X-ray diffractometer (D8 Advance; Bruker) with Cu K radiation at a voltage of 40 kV and 30 mA. The samples were scanned between 2θ = 5–60° with a scanning speed of 1°.

### 2.12. Scanning electron microscopy (SEM)

The morphology and porosity of CH-SLS beads and CH-ALG films obtained were assessed by SEM with HITACHI-S-3600H model. The films and beads were lyophilized for 24 h. The lyophilized samples were then catted into thin pieces, fixed on aluminum stubs, and sputter-coated with gold for the observation.

### 2.13. Preparation of the wound dressing

**Figure 4.**
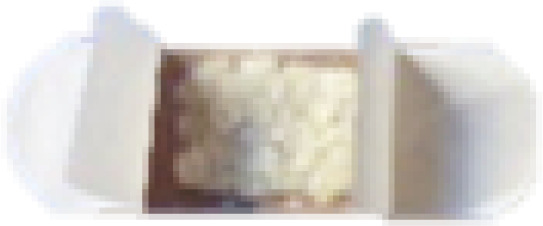
Photograph of TBF wound dressing.

The lyophilized sodium lauryl sulfate cross-linked chitosan beads containing principal active of a plant can absorb exudates while maintaining their consistency. Those beads were placed on adhesive support. Then, they were covered with the CH-ALG film which will be directly in contact with the wound. The obtained *Teucrium polium* extract-loaded CH-SLS beads and CH-ALG films (TBF) wound dressing was sterilized by absolute ethanol (70%) for 15 minutes and stored until further use (Figure 4).

### 2.14. Animal grouping

Albinos males mice were selected to carry out the experiment. The mice weighing between 17 g and 22 g were acclimatized to the laboratory environment for a period of five days. They were housed of temperature (22 ± 3°C) relative humidity (60 ± 5%) with 12 h light/dark cycle in clean plastic cages and provided with food and water *ad libitum*. Animals were divided into four groups containing 3 animals in each group.

Group I: Animals with wounds TBF-treated

Group II: Animals with wounds treated with healing ointment

Group III: Animals were covered with elastic adhesive dressing

Group IV: Animals were not treated

All animal experiments in this study were in accordance to the guidelines for animal care and use of the Directive 2010/63/EU (European Parliament and Council of the European Union, 2010) and EU Directive 86/609/EEC.

### 2.15. In-vivo wound healing study

The wound healing characteristics of the TBF-treated were evaluated using a mice model. All experiments were performed with the approval of the Institute's Animal Ethics Committee. Adults mice were anesthetized from inhalation of ethyl ether. The skin of the animal was shaved and disinfected using 70% surgical alcohol solution. The full-thickness skin wounds of the 5 cm^2^ area were prepared by excising the belly of the animals. The wound was photographed by camera phone and a sterile ruler was placed along its side to measure the wound area. Mice were then dressed with the TBF wounds dressing and then fixed with elastic adhesive dressing. For comparison, wounds treated with healing ointment were taken as positive control and treated only with elastic adhesive dressing wounds as a negative control. After treatments, the mice were housed individually in cages for 8 days starting from the day of wounding.

### 2.16. Wound rank scoring system

The macroscopic appearance of wounds in different treatment groups was blindly assessed via a wound rank scoring system that scores the degree of inflammation (redness, exudate, and swelling) [31]. Redness, exudate and swelling were evaluated according to the following scores: 0 = none; 1 = mild; 2 = moderate; and 3 = severe. The macroscopic appearance of the wound was recorded two days to for 8 days with a camera.

### 2.17. Planimetric studies

The wound-healing property was evaluated by wound contraction percentage and wound closure time. The wound size measurements taken at the time of surgery were used to calculate the percent wound contraction using the following Eq 4, where A_0_ and A_t_ were initial wound area and wound area after a time interval ‘t’. The wound area was photographed during the wound healing period at day 0 (onset of wound surgery), 2, 4, 6, and 8 by a digital camera and also was measured using a sterile ruler.

$$\begin{array}{c} Wound\;contration\left(\%\right)=\frac{A0-At}{A0}\times 100 \end{array}$$ (4)

### 2.18. Statistical analysis

Results were expressed as means ± SEM (Standard Error Mean) and statistically analyzed using Minitab.16. Statistical analysis was performed by using the one-way analysis of variance of (ANOVA) followed by Tukey's Multiple Comparison Test. Significant differences among groups in the excisional model were considered when p < 0.05.

## 3. Results and discussion

### 3.1. Preparation of beads

Ionotropic gelation of chitosan was used to prepare chitosan beads. This method is based on electrostatic interactions between the cationic chitosan and a multivalent anion SLS used as a crosslinking agent. When chitosan is dissolved in solution, the nitrogen atom on primary amine groups of chitosan is protonated by hydrogen atom from acetic acid, resulting in the formation of NH_3_^+^ groups. Unlike the cationic behavior of chitosan in acidic solution, SLS has a sulfate group (SO_4_^−^) in its anionic form. The NH_3_^+^ from chitosan and SO_4_^−^ from SLS interact through inter- and intra-molecular electrostatic interactions, inducing the formation of a sulphonate salt that is water-insoluble and precipitates as a spherical particle [29],[32],[33].

### 3.2. Beads average diameter, water content and TPC

**Table 2.** Size and biochemical characterization of chitosan beads.

	Particle size (cm)	Water content (%)	TPC (mg GAE/g)
Chitosane beads	0.18 ± 0.1	95.07 ± 0.72	61.25 ± 0.78

Table 2 shows the particle size, the water content and the total phenolic content of chitosan beads. The particle size and the water content of chitosan beads were 0.18 ± 0.1 cm and 95.07 ± 0.72%. The total phenolic content of the chitosane beads was 61.25 ± 0.78 mg GAE/g dry extract.

### 3.3. Swelling studies

Crosslinked polymers are hydrophilic materials capable to store large amounts of water between their macromolecular chains. Due to this characteristic, they are used in the biomedical field [34]. Regarding wound dressing, the degree of swelling is one of the most important properties for absorbing the exudate of the wound. Wound exudates absorption capacity of dressing was due to water absorbed by materials and water being retained in the porous structure [13]. CH-based beads are known for their ability to swell in the aqueous medium.

**Figure 5.**
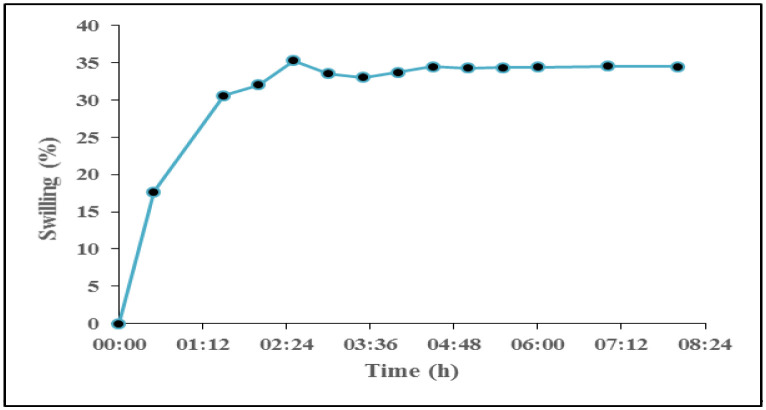
The swelling behavior of CH-SLS beads in water.

Figure 5 shows the swelling ratio of CH-SLS beads. In water, the swelling study was carried up to 8 h and the CH-SLS beads showed a swelling ratio of Qmax = 35% for 2.5 h. That value is three times higher than that of the chemical hydrogel prepared by poly (acrylamide-co-hydroxyethyl methacrylate) and five times higher than that of poly N, N-dimethylacrylamide hydrogels [35]. Hence, these beads have good swell behavior, suggesting that they have excellent water uptaken capacities. This water uptake capacity would be capable of handling the exudate volume generated from an excision wound. Fast absorption of exudates into CH-LSL beads is vital for fast wound healing because presence of a high amount of exudate slows down cell proliferation [36].

### 3.4. TPC release studies

**Figure 6.**
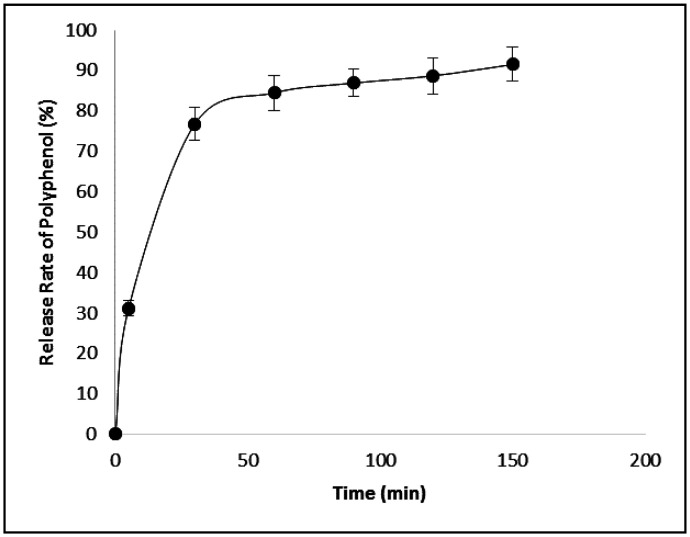
Release kinetics of the polyphenolic compounds from chitosan beads.

The release of polyphenolic compounds from chitosan beads involves simultaneous absorption of water and desorption of the compounds via a swelling-controlled mechanism. It can be seen that the total phenolic release rate increased to 91.5% at 37°C. As displayed in Figure 6, the first zone of the kinetic curve presented a fast release of polyphenols and showed an initial burst effect. The majority of the encapsulated polyphenols (more than 75%) were liberated in the first 30 min and the rest of the polyphenols were released up to 30–60 min. The rapid release of polyphenols to the medium in the first 30 min was mainly caused by the desorption of the non-incorporated polyphenols from the external region of the beads. Also, we have observed that the swelling of the polymeric matrix occurred simultaneously with the diffusion of polyphenols. In the second zone (60–150 min), the process reached a plateau and finally attained 91.5%. Similar behavior was reported by Arriola et al. [29] and Li et al. [37] who evaluated the release of phenolic content from stevia and tea respectively from sodium alginate beads.

### 3.5. Characterization of CH-SLS beads

In this study, the production of CH-SLS beads containing phenolic extracts that have been developed and characterized by chemical crosslinking using sulfate anions. CH polymer is considered as one of the best polymers used in wound dressing application due to its biocompatibility [38]. The use of sodium sulfate anions in the crosslinking of CH has been scarcely described in the literature [39].

**Figure 7.**
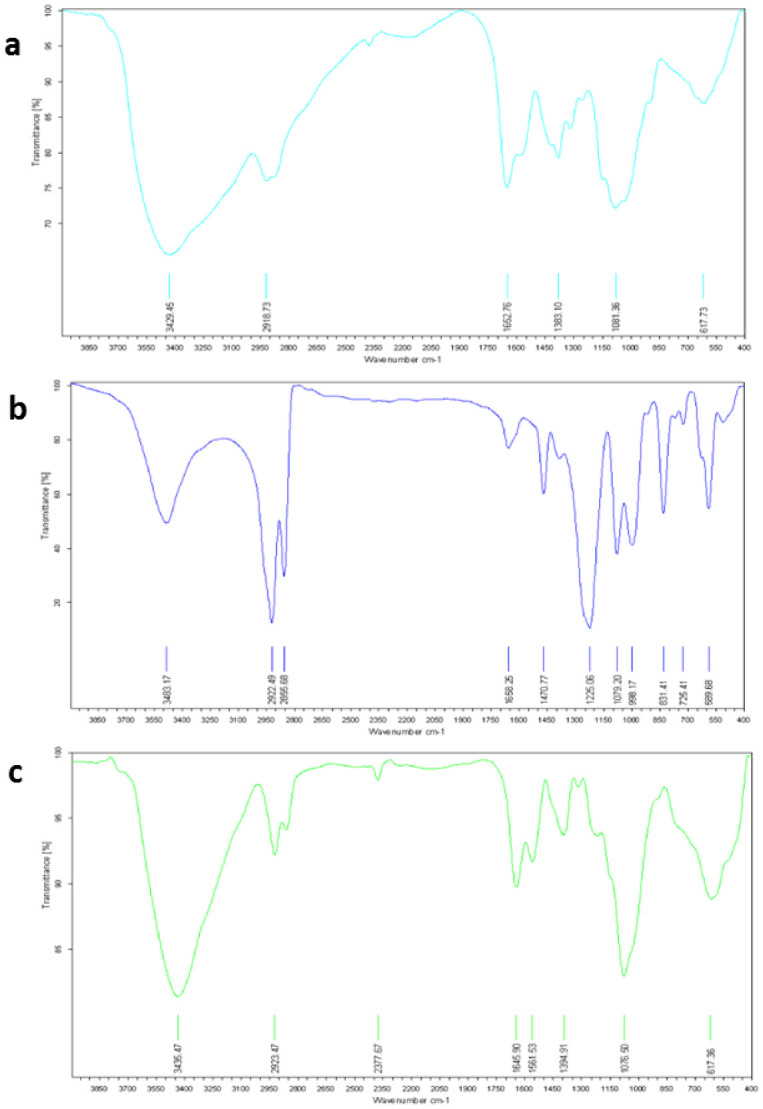
Fourier transform infrared (FT-IR). (a) chitosan (CH), (b) Sodium lauryl sulfate (SLS) and (c) Chitosan-sodium lauryl sulfate (CH-SLS) beads.

Morphology and structure characterization of CH-SLS beads were investigated by FT-IR, SEM, and XRD, respectively. FT-IR spectroscopy was used to assess the polymer chemical groups (CH, SLS, CH-LSL beads) and to investigate the formation of crosslinked networks between CH and SLS. Figure 7 shows the FT-IR spectra of CH, SLS and CH-SLS beads. From the FT-IR analysis, the characteristic peaks of CH were obtained. The broadband at 3429.45 cm^−1^ corresponded to the amine and hydroxyl groups; the peak at 2918.73 cm^−1^ was caused by -OH stretching and the absorption band of the carbonyl (C = O) stretching of the secondary amide (amide I band) at 1652.76 cm^−1^. The peaks at 1383.10 and 1081.36 cm^−1^ belong to the N-H stretching (amide III band) and the secondary hydroxyl group (characteristic peak of -CH-OH in cyclic alcohols, C-O stretch), respectively. Thus, the characteristic peak of CH at 1652.76 cm^−1^ and -S-O stretching at 1470.77 cm^−1^ of SLS were disappeared in the spectrum of CH-LSL composite hydrogel (Figure 7b). These changes confirm the formation of CH-LSL beads as a result of the interaction between the negatively charged LSL carbonyl group and the positively charged chitosan amino group. The same trend was reported in previous studies, which illustrated that CH can form microspheres with SLS [40]–[42].

**Figure 8.**
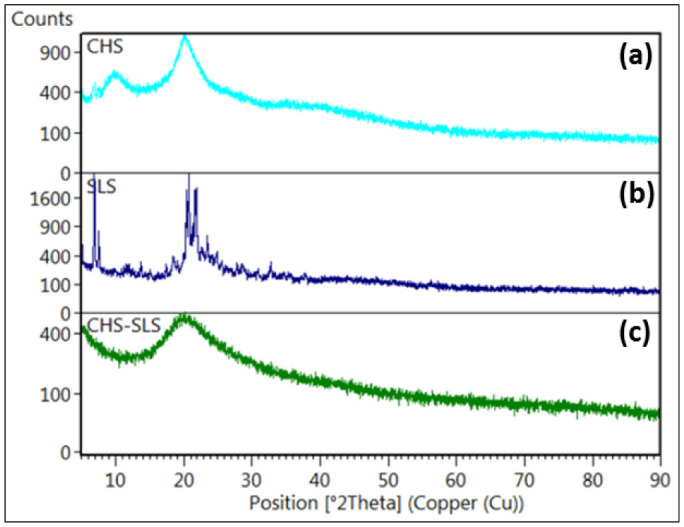
X-Ray diffraction of (a) chitosan (CH), (b) sodium lauryl sulfate (SLS) and (c) chitosan-sodium lauryl sulfate (CH-SLS) beads.

The X-ray diffraction (XRD) analysis of the CH, SLS, and CH-LSL beads are accomplished to confirm the crystallinity (Figure 8). The XRD of CH showed two peaks at 2θ = 11° and 20° which are characteristic of crystalline chitosan [43]. The highly crystalline structure of SLS is confirmed by the observed intense, narrow, and sharp peaks at 2θ = 21° and 23°. The CH-LSL beads showed a broader peak profile without an obvious crystal peak. Low-intensity diffraction peaks in CH-LSL beads are due to loss of crystallinity in the structure. Therefore, the decrease in the intensity is due to the disruption of hydrogen bonding, which resulted in an amorphous structure of the CH-SLS beads [44].

**Figure 9.**
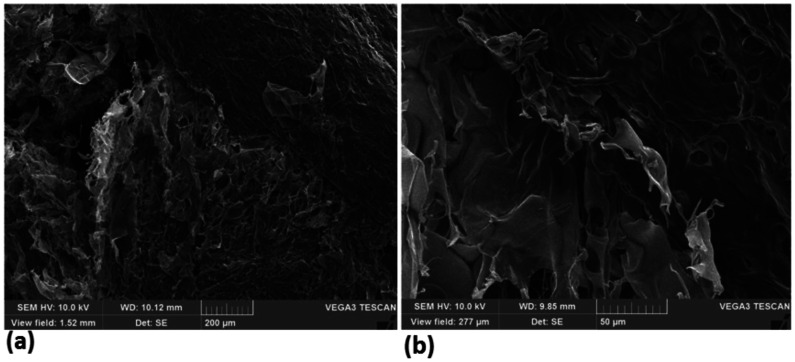
SEM micrographs of chitosan-sodium lauryl sulfate (CH-LSL) beads; (a) original magnification ×100 and (b) original magnification ×50.

Scanning electron microscopy analysis provided morphological information of the CH-SLS beads (Figure 9). The SEM showed that the obtained beads were highly porous. This structure was porous enough to absorb the excess exudates. Dehydrated beads observations show that they have a porous structure filled with water. Those observations are similar to those of hydrogels examples based on arabinoxylan (hemicellulose component) studied to understand the processes in plant cell walls [45], or hydrogels based on CH developed as biomaterials [46]. In the latter two cases, freezing at −20°C and freeze-drying of these hydrogels made it possible to observe the porosity of the hydrogel.

### 3.6. Characterization of CH-ALG films

**Figure 10.**
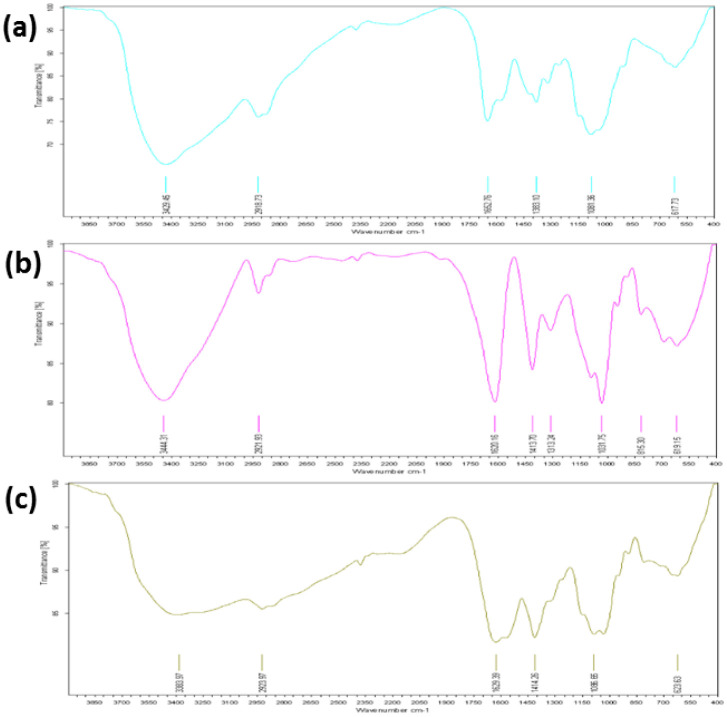
Fourier transform infrared (FT-IR). (a) chitosan (CH), (b) sodium alginate (ALG) and (c) chitosan-alginate (CH-ALG) films.

In this study, ALG, a natural and non-toxic cross-linking reagent, was used to cross-link CH. Very uniform films were obtained. The ALG crosslinked CH films were optically transparent to visible light (Figure 2). Figure 10 represents the FT-IR spectra of CH, ALG, and CH-ALG films, respectively. The FT-IR spectra suggest that the interactions between CH and ALG have occurred during the CH-ALG films formation. The characteristic peaks of ALG were observed at 1620.16 and 1413.70 cm^−1^, corresponding to two carboxyl groups (-COOH) in the molecular chain. Whereas, the carboxyl peak shifts to a higher wavelength and is observed in the films at 1629.30 cm^−1^. The FT-IR spectrum of CH also shows that the amide-I peak shifted from 1652.76 cm^−1^ to 1629.30 cm^−1^, while and the amino peak disappears after complexation with ALG. The interaction electrostatic between the carboxylate group of ALG and the amine group of CH forms a polyelectrolyte complex. The results are consistent with previous studies [47],[48].

**Figure 11.**
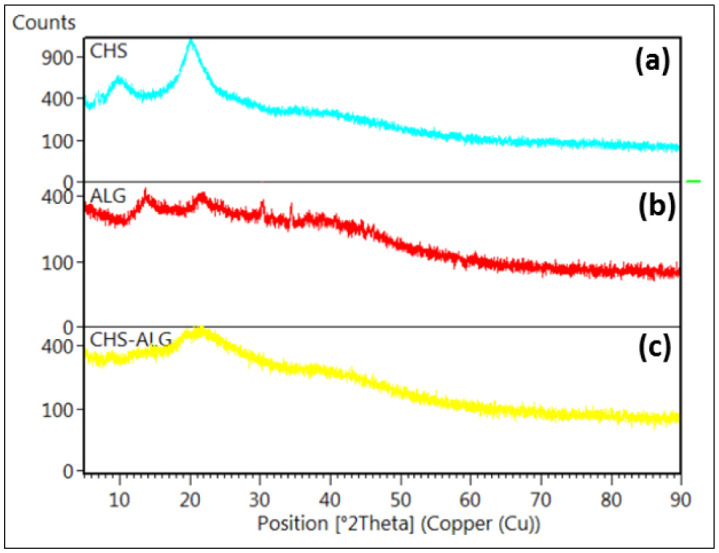
X-Ray diffraction of (a) chitosan (CH), (b) alginate (ALG) and (c) chitosan-alginate (CH-ALG) films.

XRD analysis was carried out to confirm the phase and crystallinity of the CH-ALG films. XRD of CH, ALG, and CH-ALG films are shown in Figure 11. The XRD pattern of ALG shown in Figure 11b had no crystal peak, indicating that it is an amorphous material. However, the pattern of chitosan showed the two typical diffraction peaks at 2θ = 11° and 20° (Figure 11a), which indicates that it has a crystalline structure [49]. Furthermore, the XRD pattern of the CH-ALG films had a weak broad profile without an obvious crystal peak. The formation of this film broke the hydrogen bonding between amino groups and hydroxyl groups in CH, which resulted in an amorphous structure. Similar results were obtained by Chen et al. [50], Li et al. [51], and Li et al. [52].

**Figure 12.**
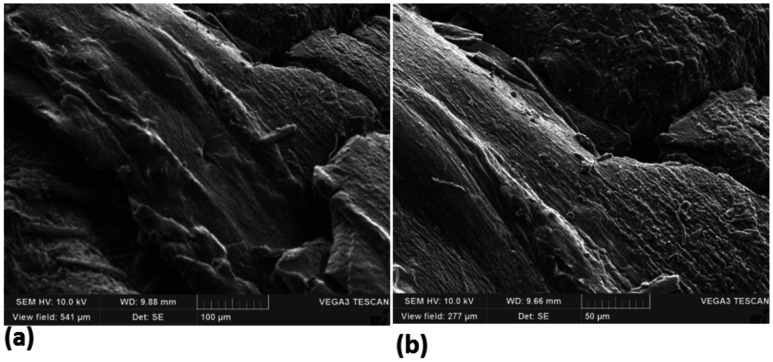
SEM micrographs of chitosan-alginate film; (a) original magnification ×100 and (b) original magnification ×50.

The surface morphology and pore distribution of CH-ALG film were examined using SEM analysis. Figure 12 shows the structure of the surface of the CH-ALG films at different magnifications. These figures indicate the fibrous and porous structure of CH-ALG films. Other researchers have also reported the same structure of CH-ALG film [48],[53],[54].

### 3.7. Film thickness, mechanical properties of CH-ALG films, and biodegradability

The thickness, mechanical properties, and biodegradability of the films were presented in Table 3. Thickness, E, and TS of chitosan-alginate films were 111.96 ± 2.54 µm, 17.06 ± 0.32%, and 9.41 ± 1.76 MPa, respectively. The Chitosan-alginate films formulated in this study could be used as a medical material because they meet a certain standard of mechanical properties [55]. Regarding biodegradation, the average value representing the weight losses of the chitosan alginate films after 14 days incubation in soil was 31.6 ± 2.73%. The chitosan used in the present study to formulate films has an average molecular weight which explains the assessed biodegradation. In fact, Halim et al. [56] reported that chitosan with high molecular weight has reduced biodegradation whereas, with low molecular weight, biodegradation rate is enhanced. Besides that, Rachmawati et al. [57] showed that chitosan film degrades naturally in soil in a period ranging from 72–87 days.

The weight loss results in the present study revealed that chitosan-alginate film is a sensitive material to the biodegrading medium. The obtained results indicate that films could be potentially degradable in natural environments.

**Table 3.** Thickness, mechanical properties and biodegradation of chitosan-alginate films.

	Thickness (µm)	Elongation (%)	Tensile Strength (MPa)	Loss weight in the soil after 14 days (%)
Chitosan-Alginate films	111.96 ± 2.54	17.06 ± 0.32	9.41 ± 1.76	31.6% ± 2.73

### 3.8. In vivo wound healing study

**Figure 13.**
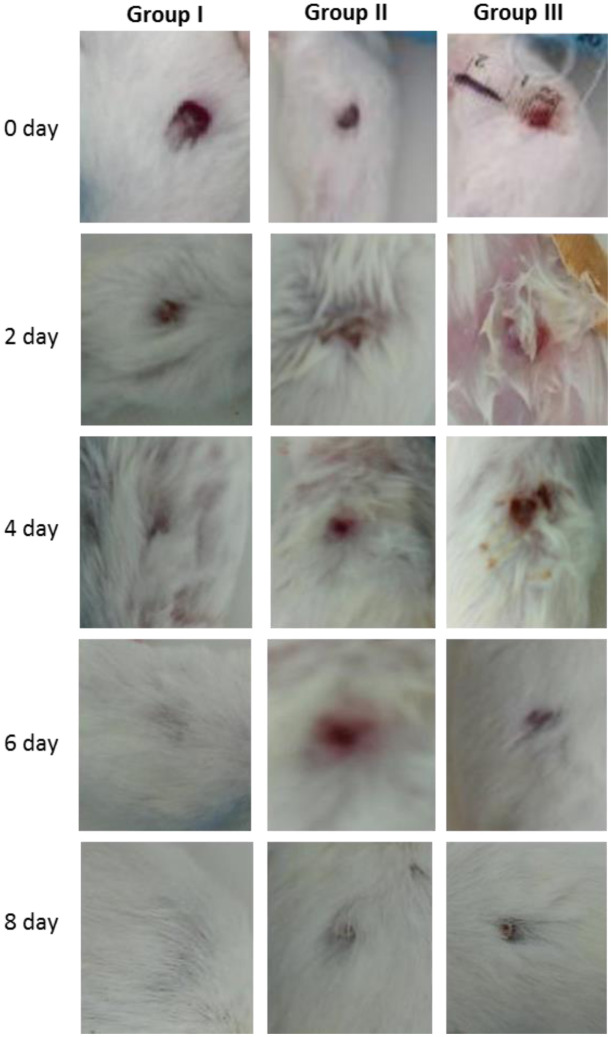
Photographs of macroscopic appearance of excision wound in TBF treated (group I), healing ointment treated (group II) or elastic adhesive dressing (group III) group at 0, 2, 4, 6 and 8 days.

The TBF wound dressing was successfully prepared from *Teucrium polium* extract-loaded CH-LSL beads and CH-ALG film. To confirm the effectiveness of the designed dressing, *in vivo* wound healing study was carried out using excision wound models. Changes in the macroscopic appearance of excision wound were checked by capturing digital images of each wound in TBF treated, healing ointment treated (positive control), or elastic adhesive dressing (negative control) groups at 0, 2, 4, 6, and 8 days. The macroscopic appearance of the wound of each group is shown in Figure 13. The healing treatment with TBF dressing was faster than the healing ointment and elastic adhesive dressing treatments at 4- and 6-days operations. At 8 days, the TBF dressings showed complete healing, while the wound treated with the healing ointment and elastic adhesive dressing had still not completely closed. The wounds treated with TBF healed more quickly than those treated with ointment and with an elastic adhesive dressing. By measuring the wound area before and after definite intervals of time, reduction in wound area was calculated.

**Figure 14.**
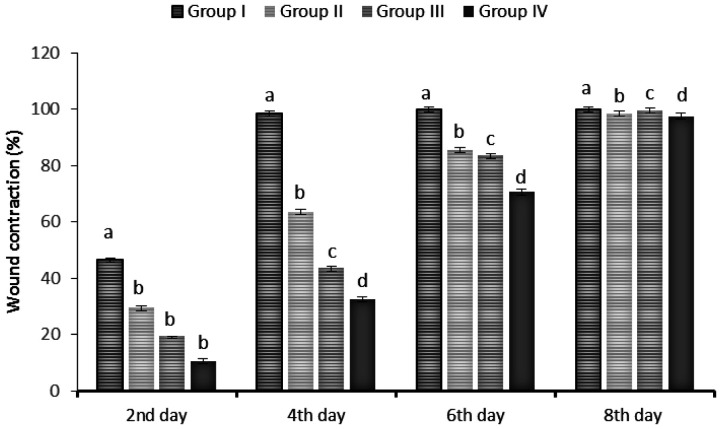
Wound contraction measurement data in TBF treated (group I), healing ointment (group II), elastic adhesive dressing (group III) or untreated group IV) groups at 0, 2, 4, 6 and 8 days. The data are presented as the means ± SD of three independent experiments; different letters on bars indicate significant differences by the Tukey multiple comparison test (p < 0.05).

Wound contraction percentage in different groups during the course of the study is shown in Figure 14. At 2 days, the contraction average of TBF wounds was about 46%, whereas the wounds treated with ointment and elastic adhesive dressing was 29% and 19%, respectively, which was statistically significant (p < 0.05). However, at 4 days, the TBF-treated wounds almost healed (98%), while 64%, 44% and, 33% of the wound contraction were achieved for the ointment, elastic adhesive dressing treated wounds and untreated wounds, respectively. Statistical analysis revealed that this difference was significant (p < 0.005). However, the wound closure was achieved only after the 8th day for the negative and positive controls wounds, which there were no statistically significant differences with TBF-chitosan wound. According to these results, healing was obviously faster and very good with TBF-chitosan dressing than the controls group.

**Figure 15.**
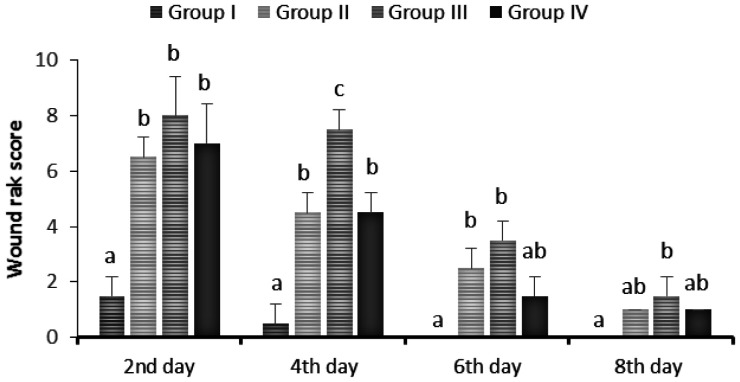
Wound rank score in TBF treated (group I), healing ointment (group II), elastic adhesive dressing (group III) or untreated group IV) groups at 0, 2, 4, 6 and 8 days. The data are presented as the means ± SD of three independent experiments; different letters on bars indicate significant differences by the Tukey multiple comparison test (p < 0.05).

The macroscopic differences in healing were quantified using a wound rank scoring system that scores the degree of inflammation. Results of the statistical analysis indicated that the wound rank scores were highly improved in the TBF-treated group I on the 4th day (p < 0.05), without any redness, exudate, or swelling in the tissues surrounding the lesion as compared to other groups (Figure 15). The data clearly indicate that the application of TBF dressing reduced skin wound rank scores. These results also showed that this dressing can keep the wound moist and was capable to absorb the wound exudate.

All the results showed that this application improves considerably the healing rate and the macroscopic wound appearance at a short delay. Khodja et al. [54] reported that the wounds treated with PVA/chitosan hydrogel healed on the 9th day, while those treated with paraffin gauze dressing and cotton gauze healed on the 16th day. Furthermore, Kim et al. [58] found that the wound healing occurred within 2 weeks when treated with chitosan hydrogel/nanofibrin composite bandages (CFBs).

The *in vivo* results clearly indicated that the wound dressing designed with *Teucrium polium* extract-loaded CH-SLS beads and CH-ALG films has many advantages to reduce the healing in an excision wound model in mice. The CH-SLS beads of such a wound dressing are beneficial for preventing wound dehydration, bacterial penetration, and also provides space to drain the accumulated exudate. In addition, the CH-ALG films could provide a moist environment at the wound site to inhibit the formation of crust. The excellent drainage ability of the TBF wound dressing is capable of handling the volume of exudate generated from the excision wounds. Indeed, the large swelling capacity of the CH beads allows the exudates emitted by the wound to be absorbed. The exudates are a source of inflammation wound [59]. Furthermore, the presence of SLS in CH-SLS beads exerts an antiseptic property, which can fit the requirement of a protective effect of a wound dressing against wound infections [60]. Then, the release of *Teucrium polium* extract from CH-SLS beads can ensure a rapid diminishing of bacteria on an excision wound. Previous studies have demonstrated the therapeutic efficacy of *Teucrium polium* as antibacterial [21], antipyretic [61], anti-inflammatory, [62] and antioxidant [23]. In addition, the wound healing property of *Teucrium polium* has already been reported due to chemical components such as flavonoids and tannin [63]. Therefore, the release of *Teucrium polium* extract from TBF wound dressing has the advantage of treating infected wounds. Our release studies suggests the effectiveness of release of water soluble polyphenolic compounds from chitosan beads to the wound site. Previous studies suggests effectiveness of a polymeric-based systems like plant polyphenol loaded alginate-chitosan composite for wound healing application [64],[65]. The results of this study have confirmed that the *Teucrium polium* extract-loaded CH-SLS beads and CH-ALG films are a promising wound dressing with enhanced wound healing.

## 4. Conclusions

In this study, we have successfully developed TBF wound dressing. This is the first report on the use of TBF as a wound dressing in an excision model in mice. The chemical structure and morphology of the beads and films were characterized by FT-IR, XRD, and SEM. The results confirmed the formation of polyelectrolyte complex between chitosan and alginate and also between chitosan and LSL. SEM results indicated that the prepared *Teucrium polium* extract-loaded Lauryl sulfate crosslinked chitosan beads have a porous structure. Furthermore, the swelling properties of those beads were analyzed in water. The optimal CH-LSL beads had a swelling ratio (Qmax = 35%) at about 2.5 h, which indicated good swelling ability. In addition, animals studies revealed that the TBF wound dressing showed a better wound healing effect in view of a rapid contraction of the wound, compared with elastic adhesive dressing and healing ointment. This dressing can keep the wound moist and was capable to absorb the wound exudate. This research opens up therapeutic prospects for skin wound care which will to be evaluated during a human's clinical trials.
